# Supplementary material for: Sexual Behaviour and Fantasies in a Group of Young Italian Cohort
Source: J Clin Med. 2021 Sep 23;10(19):4327. doi: 10.3390/jcm10194327 (PMC8509395; doi:10.3390/jcm10194327)
Supplement: Supplementary file 1 [file jcm-10-04327-s001.zip › jcm-1394661-supplementary.pdf]

SUPPLEMENTARY MATERIALS

Table S1. Sexual pleasure patterns in the all population.

**Participants, n=12590**	
**Secretly observe a naked person, Likert scale**	
**Responders, n (%)**	
1	3741 (29.7)
2	2734 (21.7)
3	2944 (23.4)
4	2270 (18.0)
5	901 (7.2)
**Secretly observe a sexual relationship, Likert scale**	
**Responders, n (%)**	
1	4568 (36.3)
2	2809 (22.3)
3	2574 (20.4)
4	1731 (13.7)
5	908 (7.2)
**Show the genitals to another person who does not expect it, Likert scale**	
**Responders, n (%)**	
1	7964 (63.3)
2	1916 (15.2)
3	1292 (10.3)
4	866 (6.9)
5	552 (4.4)
**Touch or rub against an unwilling person , Likert scale**	
**Responders, n (%)**	
1	9461 (75.1)
2	1114 (8.8)
3	791 (6.3)
4	691 (5.5)
5	533 (4.2)
**Feel pain , Likert scale**	
**Responders, n (%)**	
1	7232 (57.4)
2	2042 (16.2)
3	1733 (13.8)
4	1114 (8.8)
5	469 (3.7)
**Hit and hurt , Likert scale**	
**Responders, n (%)**	

1	8244 (65.5)
2	2002 (15.9)
3	1285 (10.2)
4	747 (5.9)
5	312 (2.5)
**Humiliated Psychologically , Likert scale**	
**Responders, n (%)**	
1	11158 (88.6)
2	642 (5.1)
3	386 (3.1)
4	237 (1.9)
5	167 (1.3)
**Humiliates Psychologically, Likert scale**	
**Responders, n (%)**	
1	10794 (85.7)
2	902 (7.2)
3	467 (3.7)
4	275 (2.2)
5	152 (1.2)
**Being tied up, Likert scale**	
**Responders, n (%)**	
1	3602 (28.6)
2	1891 (15.0)
3	2859 (22.7)
4	2486 (19.7)
5	1752 (13.9)
**Sex with animals, Likert scale**	
**Responders, n (%)**	
1	12191 (96.8)
2	230 (1.8)
3	89 (0.7)
4	59 (0.5)
5	21 (0.2)
**Sex with corpses, Likert scale**	
**Responders, n (%)**	
1	12406 (98.5)
2	103 (0.8)
3	42 (0.3)
4	20 (0.2)
5	19 (0.2)
**Dressing in clothes or underwear of the opposite sex, Likert scale**	
**Responders, n (%)**	

1	10185 (80.9)
2	1123 (8.9)
3	735 (5.8)
4	359 (2.9)
5	188 (1.5)
**Sex with people younger than 20 years against you, Likert scale**	
**Responders, n (%)**	
1	10825 (86.0)
2	657 (5.2)
3	500 (4.0)
4	351 (2.8)
5	257 (2.0)
**Sex with people older than 20 years against you, Likert scale**	
**Responders, n (%)**	
1	5608 (44.5)
2	1888 (15.0)
3	2279 (18.1)
4	1806 (14.3)
5	1009 (8.0)
**Sex with people with physical disabilities, Likert scale**	
**Responders, n (%)**	
1	11535 (91.6)
2	654 (5.2)
3	329 (2.6)
4	45 (0.4)
5	27 (0.2)
**Sex with people with mental disabilities, Likert scale**	
**Responders, n (%)**	
1	12023 (95.5)
2	369 (2.9)
3	148 (1.2)
4	25 (0.2)
5	25 (0.2)
**Exclusive attraction for a specific part of the body, Likert scale**	
**Responders, n (%)**	
1	6151 (48.9)
2	1592 (12.6)
3	2201 (17.5)
4	1586 (12.6)
5	1060 (8.4)
**Which part of the body, Likert scale**	
**Responders, n (%)**	

Breast	859 (18.9)
Genitals	423 (9.3)
Buttocks	401 (8.8)
Feet	320 (7)
Hands	300 (6.6)
Everything	651 (14.3)
Other	9636 (35.1)
**Urinating on someone, Likert scale**	
**Responders, n (%)**	
1	11277 (89.6)
2	621 (4.9)
3	395 (3.1)
4	173 (1.4)
5	124 (1.0)
**Being urinated on him, Likert scale**	
**Responders, n (%)**	
1	11461 (91.0)
2	482 (3.8)
3	329 (2.6)
4	198 (1.6)
5	120 (1.0)
**Exciting in the absence of oxygen, Likert scale**	
**Responders, n (%)**	
1	10065 (79.9)
2	1086 (8.6)
3	735 (5.8)
4	467 (3.7)
5	237 (1.9)
**Inducing lack of oxygen, Likert scale**	
**Responders, n (%)**	
1	10480 (83.2)
2	829 (6.6)
3	684 (5.4)
4	421 (3.3)
5	176 (1.4)
**Sex with more people, Likert scale**	
**Responders, n (%)**	
1	3000 (23.8)
2	2115 (16.8)
3	3412 (27.1)
4	2543 (20.2)
5	1520 (12.1)

**Observing the partner who has relationships with others, Likert scale**	
**Responders, n (%)**	
1	10741 (85.3)
2	832 (6.6)
3	560 (4.4)
4	291 (2.3)
5	166 (1.3)
**Exchange of couples, Likert scale**	
**Responders, n (%)**	
1	9135 (72.6)
2	1351 (10.7)
3	1187 (9.4)
4	597 (4.7)
5	320 (2.5)
**Blindfolded sex, Likert scale**	
**Responders, n (%)**	
1	2747 (21.8)
2	1677 (13.3)
3	3211 (25.5)
4	2853 (22.7)
5	2102 (16.7)
**Recover during sexual masturbation, Likert scale**	
**Responders, n (%)**	
1	5078 (40.3)
2	2082 (16.5)
3	2365 (18.8)
4	1869 (14.8)
5	1196 (9.5)
**Sex with pregnant women, Likert scale**	
**Responders, n (%)**	
1	10548 (83.8)
2	958 (7.6)
3	623 (4.9)
4	265 (2.1)
5	196 (1.6)
**Observed while having sex/during masturbation, Likert scale**	
**Responders, n (%)**	
1	7217 (57.3)
2	1869 (14.8)
3	1720 (13.7)
4	1054 (8.4)
5	730 (5.8)

**Sex in a public place, Likert scale**	
**Responders, n (%)**	
1	2482 (19.7)
2	2066 (16.4)
3	3246 (25.8)
4	2791 (22.2)
5	2005 (15.9)

Table S2. Contraception use in the all cohort.

Patients, n=12590	
**Do you use contraceptive methods?**	
**Responders, n (%)**	
Ring	11 (0.1)
Hormonal Contraceptive	1977 (15.7)
Natural Methods	582 (4.6)
Spiral	58 (0.5)
I Don't Use It	1571 (12.5)
I Don't Make Sex	69 (0.5)
Preservation	8311 (66.0)
Spermicides	5 (0.0)
Sterilization	2 (0.0)
**How often does condom (male or female) use to protect you from sexually transmitted infections (STIs)?**	
**Responders, n (%)**	
Never	3512 (27.9)
About 25%	1536 (12.2)
About in the middle	836 (6.6)
About 75%	2041 (16.2)
In all relationships	4665 (37.1)
**What reasons push you to NOT use sexually transmitted infections (STIs) protections?**	
**Responders, n (%)**	
I always use them	4103 (32.6)
I am sure that the partner does not have MST	5968 (47.4)
they interrupt the relationship	245 (1.9)
I trust the prtner	1633 (13.0)
I feel less pleasure	641 (5.1)

Table S3. Sexual intercourse patterns.

Patients, n=12590	
**Have you ever paid for sex?**	
**Responders, n (%)**	
Yes	492 (3.9)
No	12098 (96.1)
**Have you ever been paid for sex?**	
**Responders, n (%)**	
Yes	254 (2.0)
No	12336 (98.0)
**Partners to date, number median (IQR)**	3 (1-5)
**Do you practice passive anal sex?**	
**Responders, n (%)**	
Yes	2475 (19.7)
No	10115 (80.3)
**Do you practice active anal sex?**	
**Responders, n (%)**	
Yes	1830 (14.5)
No	10760 (85.5)
**How many penetrative sexual intercourse do you have in a month?**	
**Responders, n (%)**	
Nobody	1509 (12.0)
Less Than 4	2563 (20.4)
More Than 4	4420 (35.1)
More Than 10	4098 (32.5)
**How many non-penetrative sexual intercourse do you have in a month?**	
**Responders, n (%)**	
Nobody	2291 (18.2)
Less Than 4	2510 (19.9)
More Than 4	3809 (30.3)
More Than 10	3980 (31.6)
**How many masturbatory acts does it have in a month?**	
**Responders, n (%)**	
Nobody	825 (6.6)
Less Than 4	1930 (15.3)
More Than 4	3170 (25.2)
More Than 10	6665 (52.9)
**Longest period of sexual abstinence in your life, months median (IQR)**	4 (2-10)

**Do you reach orgasm during a relationship?**	
**Responders, n (%)**	
Never	888 (7.1)
Rarely	1906 (15.1)
Often	5181 (41.2)
Always	4615 (36.7)
**Have you ever had multiple orgasms (multiple consecutive orgasms)?**	
**Responders, n (%)**	
Yes	6053 (48.1)
No	6537 (51.9)
**Do you define yourself as a stressed person?**	
**Responders, n (%)**	
Yes	7796 (61.9)
No	4794 (38.1)
**Have had sexual experiences that I may have negatively affected my sex life (e.g. harassment, abuse, violence…)**	
**Responders, n (%)**	
Yes	2084 (16.6)
No	10506 (83.4)
**Have you ever had sex with colleagues?**	
**Responders, n (%)**	
Yes	2155 (17.1)
No	10435 (82.9)
**Have you ever cheated on your partner (now or in the past)?**	
**Responders, n (%)**	
Yes	3418 (27.1)
No	9172 (72.9)
**Do you consider yourself sexually satisfied?**	
**Responders, n (%)**	
Yes	8991 (71.4)
No	3599 (28.6)
**If you don't already have any, do you want children?**	
**Responders, n (%)**	
Yes	8471 (67.3)
No	3680 (29.2)
I already have children	439 (3.5)

Table S4. Dating app use in the all cohort.

Participants, n=12590	
**What dating sites / apps do you usually use?**	
**Responders, n (%)**	
Adotta Un Ragazzo	8 (0.1)
Annunci69	4 (0.0)
Badoo	209 (1.7)
Bakeka Incontri	5 (0.0)
Connected To Me	7 (0.1)
Grinder	104 (0.8)
Happn	17 (0.1)
Instagram	9 (0.1)
Kik	10 (0.1)
Lovoo	402 (3.2)
Meetic	146 (1.2)
Ok Cupid	9 (0.1)
Omegle	13 (0.1)
Sayhi	6 (0.1)
Tinder	836 (6.6)
Wapa	55 (0.4)
None	10750 (85.4)
**Which of these social networks do you use to meet new partners?**	
**Responders, n (%)**	
Facebook	2438 (19.4)
Instagram	3789 (30.1)
Snapchat	8 (0.1)
Telegram	65 (0.5)
Twitter	12 (0.1)
Others	20 (0.2)
None	6258 (49.7)
**What are you looking for in a dating app / site or on social media?**	
**Responders, n (%)**	
Lover	41 (0.3)
Friendship	2569 (20.4)
Occasional Partner	1443 (11.5)
Stable Partner	507 (4.0)
Virtual Relationship	57 (0.5)
I don’t use them	7973 (63.3)
**How often do you use dating / social apps / sites to look for a partner?**	

Responders, n (%)	
Never	9355 (74.3)
Few Times A Year	1745 (13.9)
About Once A Month	290 (2.3)
About Once A Week	181 (1.4)
Several Times A Month	282 (2.2)
Several Time A Week	363 (2.9)
Several Times A Day	171 (1.4)
Every Day	203 (1.6)
**What reasons motivate you to use dating or social apps / sites to look for a partner?**	
**Responders, n (%)**	
No Particular Reason	127 (1.0)
Curiosities	6 (0.0)
Discretion	153 (1.2)
It Is Fun	12 (0.1)
It Is Easier To Have Sexual Intercourse	111 (0.9)
You Can Have More Freedom	148 (1.2)
Is Easier	990 (7.9)
Greater Availability Of The Pertner	71 (0.6)
You Don't Have To Expose Yourself	434 (3.4)
You Can Meet More People	1085 (8.6)
Save Time	624 (5.0)
I Don't Use Them	8829 (70.1)
**How often do you send nude / genital photos?**	
**Responders, n (%)**	
Never	6558 (52.1)
Few Times A Year	2922 (23.2)
About Once A Month	926 (7.4)
About Once A Week	446 (3.5)
Several Times A Month	1062 (8.4)
Several Time A Week	576 (4.6)
Several Times A Day	40 (0.3)
Every Day	60 (0.5)
**How frequently do you receive nude / genital photos?**	
**Responders, n (%)**	
Never	5320 (42.3)
Few Times A Year	3623 (28.8)
About Once A Month	1129 (9.0)
About Once A Week	425 (3.4)
Several Times A Month	1247 (9.9)
Several Time A Week	656 (5.2)

Several Times A Day	82 (0.7)
Every Day	108 (0.9)
**How did you meet your current partner or the last partner you had**	
**Responders, n (%)**	
At Work	394 (3.1)
At School	860 (6.8)
Common Event (Party, Concert, Wedding, Etc.)	155 (1.2)
University	281 (2.2)
Mutual Friends	6803 (54.0)
Childhood / Family Friends	45 (0.4)
Common Hobbies (Sports, Music, Theater)	210 (1.7)
Casually	1333 (10.6)
Chat / Social / Dating App	2112 (16.8)
He Had Been My Friend For A Long Time	100 (0.8)
Online Game	12 (0.1)
On Vacation	183 (1.5)
I Don't Have A Partner	102 (0.8)
